# Sharing and Receiving Eye-Contact Predicts Mate Choice After a 5-Minute Conversation: Evidence from a Speed-Dating Study

**DOI:** 10.1007/s10508-023-02806-0

**Published:** 2024-02-20

**Authors:** Alexandra Hoffmann, Sabrina Schiestl, Philipp Sinske, Matthias Gondan, Pierre Sachse, Thomas Maran

**Affiliations:** 1 Department of Psychology, Institute of Psychology, Universität Innsbruck, Universitätsstraße 5–7, 6020 Innsbruck, Austria; 2 Entrepreneurship, Innovation and Management, Free University of Bozen, Bozen, Italy; 3 LeadershipWerk, Vaduz, Liechtenstein

**Keywords:** Speed-dating, Mutual eye-contact, Dual mobile eye-tracking, Human mate selection, Face-to-face interaction

## Abstract

In popular narratives, the first date with a potential mate often centers on their gaze as embodiment of interest and attraction. However, evidence is still lacking on the role of eye-contact as a potent signal in human social interaction in the context of dating. In addition, behavioral mechanisms of mate selection are not well understood. In the present study, we therefore examined mutual eye-contact and its influence on mate choice by applying dual mobile eye-tracking during naturalistic speed-dates. A total of 30 male and 30 female subjects attended four speed-dates each (*N* = 240). Subjects were more likely to choose those dating partners with whom they shared more eye-contact with. In addition, perceived attractiveness played an important role for mate choice. Interestingly, receiving but not giving eye-contact also predicted individual mate choice. Eye-contact thus acts as an important signal of romantic attraction when encountering a dating partner.

## Introduction

Imagine you meet someone for the first time. The two of you talk for about five minutes; afterward, you decide whether you want to meet again. What drives your decision? Anecdotes from social media or newspapers repeatedly describe that eye-contact plays a decisive role (e.g., Catron, 2015; Varina, 2022). The legend of “love at first sight” probably persists for this reason. Actually, research describes eye-contact as a natural and indispensable part of communication (Grossmann, 2017; Risko et al., 2016). More specifically, we often use eye-directed gaze to signal liking or attraction (Fullwood, 2007); vice-versa eye-contact affects likeability and attractiveness (Hietanen, 2018; Mason et al., 2005). Furthermore, eye-contact plays a role during courtship (e.g., for making someone approach you) (Apostolou & Christogorou, 2020; Moore, 1985; Walsh & Hewitt, 1985). Although strong evidence points toward eye-contact as a driving force behind signaling interest and choosing a potential mate, it is still unknown whether it influences mate choice. To date, studies on human mate selection mainly rely on self-report measures. Thus, there is only sparse evidence for behavioral predictors of mate choice. Interestingly, one of the most potent non-verbal signals i.e., mutual eye-contact (Senju & Johnson, 2009) has not been measured unobtrusively during romantic interactions.

We aim to bridge this gap by investigating mutual eye-contact during speed-date conversations. In our study, subjects were talking to an unknown person of the other sex, while we continuously tracked eye-movements of both dating partners with two pairs of mobile eye-tracking glasses. Therefore, our research is designed to measure naturalistic gaze behavior between individuals and thus the dual function of eye-gazing in the dating context. We further unravel the role of eye-contact in mate choice by connecting mutual eye-contact with the probability of choosing a mate and therefore investigate whether shared eye-contact acts as a predictor of mate choice. Choosing a mate might be predictable in social interactions beyond subjective perceptions by an objectively measurable communicative signal.

Mate choice is one of the most vital decisions for sexual reproduction. The evolutionary view mainly focused on identifying sexual cues such as height, facial symmetry, and scent to predict mate choice (Miller & Todd, 1998). However, not only physical appearance, but also the responding to external signals constitutes a fundamental mate choice mechanism (Rosenthal & Ryan, 2022). Individuals favor signals that are easily detectable and sensorily stimulating (Ryan & Keddy-Hector, 1992); such signals are most strongly perceived during immediate social interaction (Helminen et al., 2011; Jarick & Bencic, 2019). Therefore, signals like verbal proficiency (Lange et al., 2014), voice pitch (Pisanski et al., 2018), and even body movements as well as language (Renninger et al., 2004) has been observed to play a role in mate choice.

One of the most potent signals in human social interactions and thus also in the encounter with potential mates is eye-gaze (Grossmann, 2017; Risko et al., 2016). It is easily detectable (e.g., Kobayashi & Koshima, 1997), stimulating (e.g., Hietanen, 2018), designed to communicate (Emery, 2000), and drives the interaction with our social encounters (e.g., Maran et al., 2021). Although there is a large amount of literature on mate selection and cues related to it, the dynamics of eye-gaze between two individuals have been neglected so far. Nonetheless, eye-gaze fulfills a variety of functions during social interactions like gathering information, signaling attention, and drawing conclusions about other people’s mental state (Baron-Cohen, 1997; Emery, 2000; Kobayashi & Hashiya, 2011). Here, the so-called duality of eye-contact is of great importance i.e., the eyes both signal and perceive information (Gobel et al., 2015). In addition, gaze direction from others in our environment is an important cue that captures our attention. In social encounters, gaze toward others can indicate that we are interested in them. Conversely, being looked at elicits positive emotions (Hietanen, 2018), while it also creates more intimacy and less uncertainty (Croes et al., 2020). This, consequently, may trigger approach intentions toward the person from whom the eye-contact originates (Walsh & Hewitt, 1985). Direct gaze also leads to elevated perceived likability (Mason et al., 2005), charisma (Maran et al., 2019), and attractiveness (Ewing et al., 2010). Indeed, early experimental lab studies, where pairs were told to sit quietly and gaze at the other person’s eyes or hands, found a positive effect of mutual eye-contact on romantic interest (Kellermann et al., 1989; Williams & Kleinke, 1993). While there are a number of studies on naturalistic gaze behavior in dyadic interactions (Broz et al., 2012; Guy & Petrovez, 2023; Hessels et al., 2017; MacDonald & Tatler, 2018; Mayrand et al., 2023; Rogers et al., 2018), naturalistic gaze behavior between two individuals in the dating context has never been examined. Thus, the goal of our study was measuring a behavioral indicator for mate choice by applying dual mobile eye-tracking during natural dating interactions.

In romantic encounters with potential partners, eye-contact might serve as a signal that sends information and influences the recipient’s behavior (Gobel et al., 2015). Moreover, eye-contact acts as a clearly visible signal of interest (Hietanen, 2018), approach (Walsh & Hewitt, 1985), and attraction (Williams & Kleinke, 1993). In consequence, it shapes the recipient’s impression of the sender as more attractive (Kampe et al., 2001), charismatic (Maran et al., 2019), and likeable (Mason et al., 2005). Therefore, we argue that the duration of simultaneous attention toward each other’s eyes is a predictor for individual mate choice. We hypothesize that the more mutual eye-contact between two individuals during a speed-date, the higher the probability of choosing the encounter as a potential mate. In addition, we also test whether giving and receiving eye-contact predict mate choice. As earlier studies on naturalistic gaze behavior in dyadic interactions show that eye-contact is subjectively overrated (Rogers et al., 2018, 2019), we argue that subjectively perceived and objectively measured mutual eye-contact are not related with one another, therefore replicating earlier results and strengthening our mobile eye-tracking approach in a speed-dating study. As Rogers et al. (2019) demonstrated that an audience perceives someone as making eye-contact even when they are just looking at the face area i.e., the mouth or forehead, we additionally try to predict mate choice by mutual face-contact.

## Method

### Participants

Our sample was an ad-hoc sample; as we applied a multilevel design, where we observed naturalistic behavior during conversations, we could not compute a classic power analysis. Therefore, we aimed at a similar sample size as a previous study (Pisanski et al., 2018; *N* = 30), which investigated verbal proficiency as a predictor of desirability (short-term and long-term mating). Their study computed linear mixed models with maximum-likelihood estimation and found significant effects in both male and female subjects.

We recruited 60 young adults (30 female, 30 males; age ranging from 19 to 32 years with *M* = 23.1; *SD* = 2.8) that participated voluntarily in four speed-dates each, resulting in a total of 240 dyadic speed dating interactions. Due to bad data quality, that is, less than 80% of gaze samples, we excluded three speed-dates from coding and further statistical analyses, resulting in a final sample of 237 interactions. Due to the pandemic, we decided to work with pre-assigned dating partners (4 female, 4 males; age ranging from 21 to 30 years, *M* = 23.1 and *SD* = 3.5). Those dating partners were randomly chosen and unaware of the research hypothesis. The only selection criterion was that they were single at the time and motivated to participate in a series of about 30 speed-dates each. For hygienic reasons it was not allowed to have more than two people in the room at the same time, so we could not perform a round-robin design at the time of the study. Moreover, we only had two pairs of eye-tracking glasses available, so we could only conduct one speed-date at a time. All participants and dating partners were single at the time of the study, no one was married or had children; 57 participants indicated to be heterosexual, while 3 were bisexual. Participants as well as their dating partners were all European, the mean monthly income was about 600€ (*SD* = 688); the sample mainly consisted of students (*N* = 59).

### Procedure

For the purpose of this study, participants were invited to take part in a speed dating procedure, where they would attend four different speed-dates. Recruitment took place via round mail, flyers and spontaneous offering of the possibility to be part of a speed dating session on the university campus. During each of those speed-dates, they would get to know a person of the other sex briefly for about five minutes. After the time has elapsed, participants as well as their dating partners confidentially indicated whether they would like to see their counterpart again or not. The wording of this question was as follows: “Would you be interested in seeing this dating partner again after the speed-date event?” and subjects answered “yes” or “no”. The answer to this question is defined as our subjects’ actual mate choice (see also Jauk et al., 2016; Todd et al., 2007). This kind of setting has been applied in numerous studies to determine factors that influence romantic interest during the first encounter between two unfamiliar individuals (e.g., Back et al., 2011; Luo & Zhang, 2009; Tidwell et al., 2013). To simultaneously record eye-movement patterns of the two speed dating partners, we used two pairs of *Tobii Pro Glasses 2* (Tobii AB, Sweden; sampling rate 50 Hz). In our study, both dating partners were seated across from each other at 1 m distance with a table between them. Pairs were not given a topic to talk about but had free choice to ensure that the conversation was as natural as possible. Subjects were unaware that their dating partners were pre-assigned. Before starting the speed-date, subjects as well as dating partners were equipped with the eye-tracking glasses. After having completed the calibration procedure for both interaction partners in separate rooms, they were seated across each other, and we performed a clap to signal the start of the conversation. Dating partners waited in a separate room and were then brought into the subject’s room for their respective date. In total, the speed-dating session took about 1 h per subject, with four speed dates being prepared and carried out. In between the dates, subjects as well as dating partners filled out a short questionnaire; after that the eye-tracker was calibrated again for the next date.

### Measures

After each speed-date, subjects as well as dating partners indicated whether they would like to see the other person again (see Asendorpf et al., 2011). In addition, they were asked to rate their dating partners in terms of physical attractiveness on a scale ranging from 1 (very unattractive) to 10 (very attractive). Finally, we asked them to indicate how much mutual eye-contact they personally felt existed throughout the conversation on a scale ranging from 0 to 100% (10% steps; see also Rogers et al., 2018). As our sample consisted of 30 male and 30 female participants with a relatively homogenous age distribution, we did not include age as a control variable in our statistical models.

In research on mate selection, attractiveness has widely been discussed as a crucial factor influencing mate choice (Thornhill & Gangestad, 1999). Therefore, studies on romantic attraction repeatedly found perceived attractiveness to be one of the strongest predictors of mate choice (e.g., Apostolou & Christogorou, 2020; Asendorpf et al., 2011; Jauk et al., 2016; Luo & Zhang, 2009), both in women and men. The more attractive men are, the more short-term sexual partners they have, while more attractive women have more long-term sexual partners (Rhodes et al., 2005). Human attractiveness evolved because of mate preference for healthy and fertile mates (Symons, 1979), and research showed that attractiveness is highly related with the chance for reproduction (e.g., Fink et al., 2006; Weeden & Sabini, 2005). Sexual dimorphism is most important in determining attractiveness in both men and women (see Mogilski & Welling, 2017). While in women, attractiveness is mainly judged by facial symmetry (e.g., Perrett et al., 1999), averageness, and youth (Symons, 1979), the context of the relationship sought is quite important in judging male attractiveness (Little et al., 2002).

In addition, attractiveness has been shown to draw attention and lead to more eye-contact (e.g., Leder et al., 2016; Mitrovic et al., 2018; Valuch et al., 2015; Van Straaten et al., 2010). Therefore, we control for perceived attractiveness in our statistical models.

#### Coding Gaze Data

To manually code gaze behavior, we used specialized behavioral coding software that allows synchronous playback and manual coding of two audiovisual files (*Mangold Interact*, Germany; https://www.mangold-international.com/en/software/interact). Eight independent raters coded gaze behavior manually frame by frame based on gaze recordings using the *Mangold Interact Software*, which allows watching the footage in real-time while displaying a superimposed circle depicting the calculated gaze position and defining the beginning and end of each gaze event. To examine participants’ eye-movement patterns, we exported the Tobii glasses footage as.mp4 files using Tobii Pro Lab (Tobii AB, Sweden). These audiovisual recordings entail an eye-tracking overlay (red circle), which depict an individual’s attentional focus at any given time point. We used the standard parameters by the Tobii software before exporting our recordings (Rogers et al., 2018). Specifically, we applied the Tobii I-VT fixation filter, which interpolated missing points in the recordings with a maximum gap length of 75 ms. Noise was reduced by moving median with a window size of 3 samples. The maximum time between fixations was 75 ms and the maximum angle between fixations was 0.5°. The minimum fixation duration was 60 ms. The filter did not remove blinks. Tobii does not provide the margin of error; therefore, we cannot report on this. As we calibrated the eye-tracker before each date and the date took place in a seated position, we would argue that the error is rather low. In addition, the eye-tracker was affixed to the head with a head band, so that even when the head was moving, the eye-tracker stayed in the same position.

**Fig. 1:**
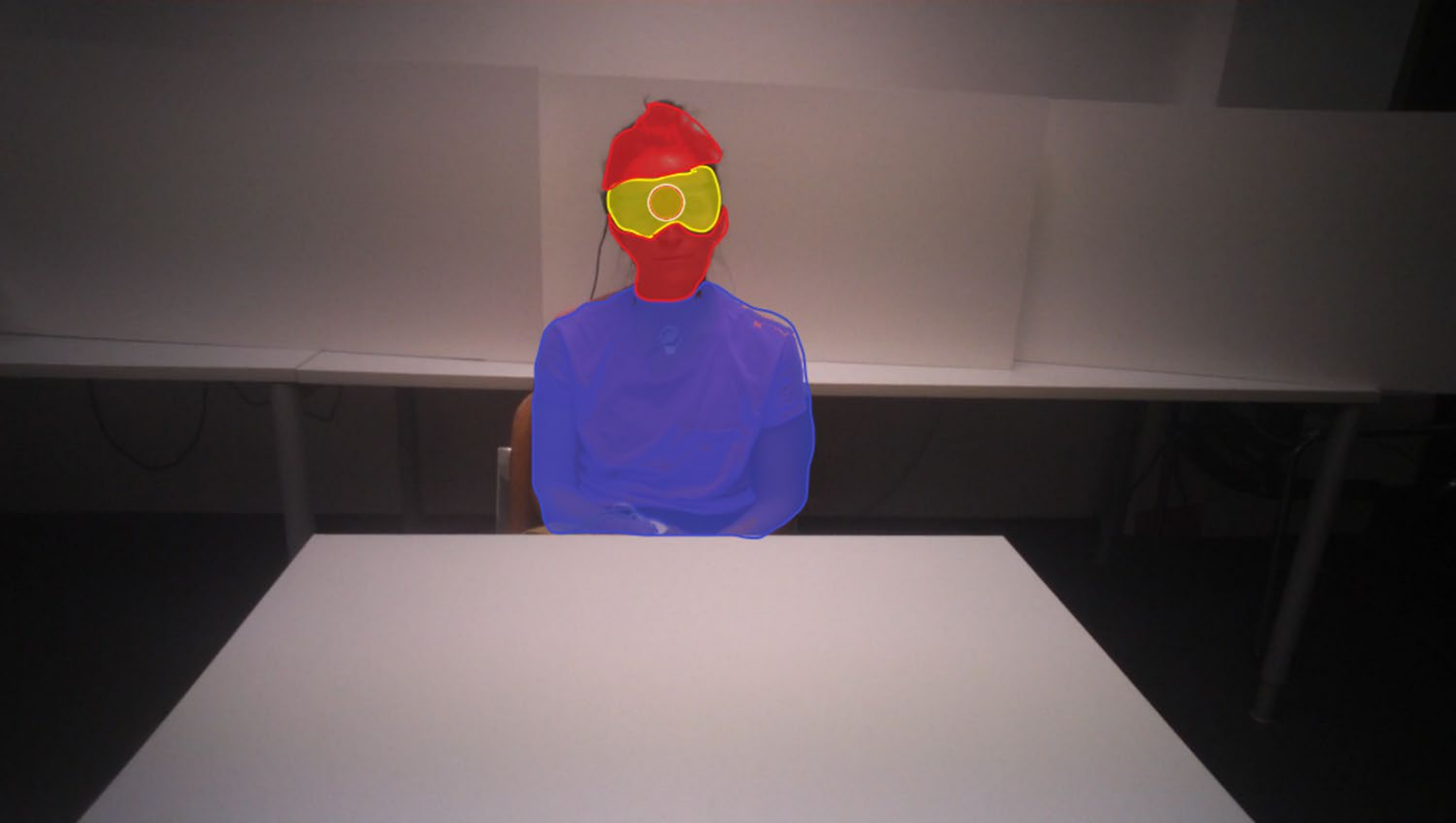
Example of AoIs during the speed dating conversation showing **a** an example view of the visual field of one of the dating partners during their interaction and **b** visualization of the AoIs used to analyze relevant gaze points during the date (red = face area, yellow, eyes area, blue = body area, white and black parts = background area). Note that we did not apply AoIs on the videos, but coders manually coded the relevant AoIs (Color figure online)

Pairs of videos were opened using the *Mangold Interact software*. The use of a clap signal enabled the recordings to be played back in-sync after adjusting start times appropriately. An individual’s gaze behavior was manually coded according to the location being fixated upon at any point in time for two different on-face locations (eyes, other-face) and off-face locations (body, background). ‘Other-face’ refers to the cheeks, mouth, jaw and forehead areas of the face, essentially any spot not covered by the ‘eyes’ location. ‘Eyes’ included both eyes and the area in-between and around the eyes (see Fig. 1). When a participant blinked, this was not included in the code. As an additional code, we combined “eyes” and “other-face” to “face”, thus covering the full-face area. The software also allowed us to quantify the extent and timing of mutual eye-gaze/face-gaze (i.e., both partners simultaneously looking at each other’s eyes/face) by applying a co-occurrence filter across the two videos of each interaction. For all our locations (eyes, other-face, face, body, and background) as well as the co-occurrence of eye- and face-gazing, we exported the number of fixations as well as the total fixation duration across whole interaction. As both parameters are highly correlated with each other (*r* = 0.91, *p* < 0.001 for count and duration of mutual eye-gaze; see also Table 4 for correlations between unidirectional eye-tracking parameters), we decided to focus on the duration parameters in our results section (see Maran et al., 2022 for a similar approach).

Coding the video of one video took about 4 h on average, so for our 60 subjects × 4 speed-dates × 2 recordings = 480 videos, our coding team spent about 2000 h of manual coding. Three interns and five master students coded those eye-tracking recordings. All coders were thoroughly trained to minimize subjectivity and ensure a common standard when coding gaze events that lay between two areas of interest. We assessed inter-rater reliability by letting our coders code the same 7 dyadic 5-min conversations (14 videos in total). Cohen’s Kappa was calculated using the Interact software and was found to be satisfactory (Kappa ranging from 0.69 to 0.86) for fixation coding. In addition, all videos were double-checked by two different raters, who corrected manual coding where necessary.

### Statistical Analyses

The primary dependent variable was the binary choice variable, namely, whether the subject reported the wish to see their dating partner again. Note that we did not consider matches as an outcome, as there were too few matches within our sample (4%), so the multilevel regression model would not converge. Also, dating partners were pre-assigned and showed a different choice behavior than subjects (see Table 1). Therefore, we considered only subjects’ choice behavior in our statistical models. We applied a multilevel logistic regression to predict subjects’ mate choice based on the duration of mutual eye-contact, with gender as a categorical covariate and subjects as random intercept. In a second step, we tested whether the duration of mutual eye-contact explained their choice behavior beyond mutually perceived attractiveness between the dating partners, computed as a mean score of the attractiveness ratings of both dating partners. We repeated the first analysis with mutual face-gaze to test whether the effect is specific for the eye region. For both analyses, we computed separate models for male and female subjects that we adjusted for dating partner. Effects of our analyses are reported as odds ratios (ORs) with their respective 95% confidence intervals. An OR = 1.35 for mutual eye-contact duration indicates that if the duration of the mutual eye-contact increases by one minute, the chance for mate choice increases by 35%. All statistical analyses were performed in R 3.6.1 (R core team, 2023).

**Table 1:** Choice behavior in subjects and their pre-assigned dating partners

Choices	Subjects		Dating partners	
	Male *N* (%)	Female *N* (%)	Male $\overline{M}$ (range)	Female $\overline{M}$ (range)
0	8 (26.67%)	14 (46.67%)		
1	7 (23.33%)	8 (26.67%)		
2	8 (26.67%)	6 (20%)	6 (1–12)	5 (1–8)
3	6 (20%)	1 (3.33%)		
4	1 (3.33%)	1 (3.33%)		

For subjects, we provide the number of subjects that chose 0, 1, 2, 3 or all 4 of their dating partners. For the pre-assigned dating-partners, we provide the mean value of the number of subjects they chose (max = 30)

## Results

On average, subjects indicated that they would like to meet their dating partner a second time (“choices”) in 72 speed-dates (30%), with the choice frequencies ranging from 0 to 4 (see Table 1). Each dating partner attended 30 speed-dates in total; not unexpectedly, their choice frequency was lower (43 choices, 18%). A total of only 10 mutual choices (4%, “matches”) were recorded in the 240 speed-dates. Gaze behavior is summarized in Table 2 for the frequency of eye-contact, their total duration, and the time relative to the entire speed dating procedure (about 5 min.). In line with earlier results (Rogers et al., 2018), we observed substantial differences between self-reported (*M* = 60%) and objectively measured eye-contact (*M* = 8.25%; see Table 2), and the correlation between these variables, although significant, was rather low (*r* = 0.15, *p* = 0.020).

**Table 2:** Eye-contact as measured by the eye-tracking glasses

	Total count (Range)	Total duration (Range)	Relative time (%)
Given eye-contact	288.57 (4–635)	90.64 s (0.56–250.60 s)	30.63
Received eye-contact	236.83 (0–655)	87.66 s (0.00–280.28 s)	29.22
Mutual eye-contact	125.00 (0–551)	24.76 s (0.00–111.56 s)	8.25

### Predicting Mate Choice with Mutual Eye-Contact

Results of our multilevel regression model indicated threefold chances for a positive mate choice with each additional minute of mutual eye-contact (OR = 2.80, 95% CI from 1.22 to 6.32, *p* = 0.015), with female subjects tending to be choosier i.e., they were less likely to say yes to want to seeing their dating partner again (OR = 0.43, *p* = 0.068). We further conducted a sensitivity analysis with mutually perceived attractiveness as an additional covariate in order to determine whether eye-contact or attractiveness is the stronger predictor for mate choice. In this analysis, both mutual eye-contact (OR = 2.70, 95% CI from 1.15 to 6.34, *p* = 0.023) and mean perceived attractiveness (OR = 1.33, 95% CI from 1.01 to 1.75, *p* = 0.046) predicted mate choice; again, women were choosier than men (OR = 0.38, *p* = 0.049). By contrast, neither mutual face-gaze (OR = 1.49, *p* = 0.124) nor self-reported eye-contact (OR = 1.24, *p* = 0.085) predicted subjects’ mate choice. Gender-specific analyses with covariates for mutually perceived attractiveness and dating partner as well as a random intercept for participant did not result in any specific effects for male subjects (attractiveness: OR = 0.88, *p* = 0.524; mutual eye-contact: OR = 1.46, *p* = 0.502), while for female subjects, attractiveness seemed to play a stronger role (OR = 2.40, 95% CI from 1.11 to 5.22, *p* = 0.027) than mutual eye-contact (OR = 2.90, *p* = 0.261) for their mate choice Table 3.

**Table 3:** Descriptive statistics (*M* = mean value, *SD* = standard deviation) of eye-tracking parameters for each area of interest (AoI), displayed separately for male and female subjects

	Male subjects *M *(*SD*)	Female subjects *M *(*SD*)
Eyes count	291.38 (116.62)	285.75 (142.15)
Eyes duration	106.78 (70.32)	76.86 (50.22)
Head count	208.17 (93.80)	243.74 (107.13)
Head duration	51.32 (35.01)	51.45 (32.03)
Body count	41.03 (53.55)	46.74 (56.36)
Body duration	9.55 (15.66)	9.13 (13.61)
Background count	109.73 (72.96)	120.41 (79.18)
Background duration	24.80 (16.77)	23.37 (19.36)

Last, we checked whether perceived attractiveness was related to eye-tracking parameters; interestingly, mean perceived attractiveness was lightly correlated with mutual face contact (*r* = 0.16, *p* = 0.020), but not with mutual eye-contact (*r* = 0.10, *p* > 0.05).

### Predicting Mate Choice with Given and Received Eye-Contact

In an additional exploratory analysis, we were interested in whether one-sided eye-gazing could predict mate choice in our subjects just as well as mutual eye-gaze. Therefore, we calculated another model with subjects’ duration of eye-gaze and gender as predictors and their mate choice as the dependent variable. Surprisingly, subjects’ eye-gazing toward their dating partners did not predict their mate choice (*p* = 0.56).

In contrast, receiving eye-contact from their dating partners indicated threefold chances for a positive choice with each additional minute of received eye-contact in our subjects (OR = 1.72, 95% CI from 1.21 to 2.43, *p* < 0.01). We further conducted a sensitivity analysis with an additional covariate for perceived attractiveness. In this analysis, both received eye-contact (OR = 1.72, 95% CI from 0.97 to 2.05, *p* < 0.001) and perceived attractiveness (OR = 1.35, 95% CI from 0.97 to 2.05, *p* < 0.001) predicted subjects’ mate choice. Gender-specific analyses with covariates for attractiveness and dating partner as well as random intercept for subject showed that while in women attractiveness (OR = 2.53, 95% CI from 1.12 to 5.71, *p* = 0.026) was the sole predictor for their choice, in men receiving eye-contact (OR = 1.94, 95% CI from 1.14 to 3.30, *p* = 0.015) mainly influenced their choice behavior Table 4.

**Table 4:** Correlations between eye-tracking parameters of the evaluated areas of interest

	1	2	3	4	5	6	7
1. Eyes count	–						
2. Eyes duration	0.73***	–					
3. Head count	− 0.23***	− 0.37***	–				
4. Head duration	− 0.28***	− 0.19**	0.84***	–			
5. Body count	− 0.53***	− 0.48***	0.42***	0.43	–		
6. Body duration	− 0.47***	− 0.39***	0.33***	0.38	0.95***	–	
7. Background count	− 0.41***	− 0.42***	− 0.11	− 0.15	0.05	0.00	–
8. Background duration	− 0.35***	− 0.27***	− 0.15*	− 0.11	0.04	0.02	0.94***

*p < 0.05, **p < 0.01, ***p < 0.001

Again, we checked whether unidirectional eye-contact variables and perceived attractiveness were related to each other; interestingly, subjects gaze toward their dating partners’ eyes was not related to their attractiveness ratings of the same (see Table 5). The eye-gazing subjects received from their dating partners was lightly related to their attractiveness perception, though.

**Table 5:** Full sample correlations between attractiveness ratings for dating partners and unidirectional fixations toward the eye region

	1	2	3	4
1. Partners*’* attractiveness	–			
2. Subjects*’* eyes duration	− 0.04	–		
3. Subjects*’* eyes count	− 0.02	0.73***	–	
4. Partners*’* eyes count	0.14*	− 0.05	− 0.04	–
5. Partners*’* eyes duration	0.15*	− 0.07	− 0.07	0.87***

Results of this analysis were similar for both male and female subjects; we therefore report correlations across the full sample

*p < 0.05, **p < 0.01, ***p < 0.001

## Discussion

Going on blind dates is a very common thing nowadays (Economic Times Online and Agencies, 2023). Yet, to date, we know little about behavioral dynamics that might influence interpersonal attraction between individuals meeting for the first time. In the present study, we shed light on the effects of mutual eye-contact on attraction between two individuals meeting in a dating context for the first time. In support of our hypothesis, mutual eye-contact predicted individual mate choice beyond perceived attractiveness. Our results show that subjects sharing more eye-contact with their dating partner, were more likely to want to see their dating partner again. Thus, keeping eye-contact seems to have a strong influence on being attracted to someone. Although participants tend to misjudge the duration of eye-contact (see also Rogers et al., 2018), we could show that only eye gaze, but not face gaze, has an influence on mate choice. This in turn reinforces our assumption that specifically eye-contact plays a fundamental role in mate choice. Additional analyses further revealed that receiving but not giving eye-contact predicts mate choice. Our findings add a piece to the puzzle of how behavioral indicators influence mate choice behavior. This is especially important as to date the evidence for behavioral cues in contrast to self-report measures is sparse (e.g., Lange et al., 2014; Pisanski et al., 2018; Renninger et al., 2004).

Two explanatory approaches aligned with the functions of interpersonal eye-contact offer an answer to these findings. First, eye-contact might be a flirting tactic worth applying to court a stranger, as real eye-contact by others triggers positive arousal, and thus, the counterpart is perceived more positively (Hietanen, 2018). Another person’s gaze is a powerful social cue, as the direction of their gaze regulates the interaction, but also expresses intimacy and social control (e.g., Croes et al., 2020; Kleinke, 1986). Direct gaze is associated with more positive evaluations and liking (e.g., Kuzmanovic et al., 2009; Mason et al., 2005) as well as more pleasant feelings (e.g., Chen et al., 2017; Uono & Hietanen, 2015).

In terms of our findings, this might imply that subjects reciprocated eye-contact with those dating partners they chose to shape their impressions about themselves (Maran et al., 2019). Our results highlight the fact, that sharing more eye-contact has a positive influence on individual mate choice. This indicates that the positive experience of being looked in the eyes has a direct behavioral implication. We assume that sharing eye-contact with your interaction partner has a strong impact on your decision whether you would choose to see that person again or not.

Second, eye-contact is perceived as charismatic (Maran et al., 2019, 2020), and thus might have led subjects to be more attracted to those dating partners with whom they had more eye-contact. Maran et al. (2019) suggest that the charismatic effect of eye-contact induced in others is rooted in the self-referential processing that perceived eye-contact induces (Conty et al., 2010; Ho et al., 2015). In other words, when being looked at we feel “touched” from those who are looking at us. Indeed, eye-gaze acts like a pointer, just as calling someone’s name (Kampe et al., 2003). Thus, if an individual receives more eye-contact from their dating partner, they might feel more involved and create a more personal bond with their counterpart. Translated onto our design, we are more likely to choose those dating partners, with whom we shared more eye-contact, and also from who we received more eye-contact. To conclude, sharing eye-contact while you get acquainted with someone might be important to create intimacy and bond with each other.

In addition to eye-contact, attractiveness could also explain mate choice, confirming results of earlier studies on romantic attraction (e.g., Asendorpf et al., 2011; Jauk et al., 2016; Luo & Zhang, 2009). Interestingly, attractiveness seemed to play a more important role for female than male subjects. The more attractive their dating partner, the more likely subjects were to want to see them again. Thus, we assume that attractive dating partners are more likely to be chosen for another date, as attractiveness is an important indicator of health and reproduction, which form fundamental mechanisms during mating (e.g., Fink et al., 2006; Weeden & Sabini, 2005). Kampe et al. (2001) assumed that attractiveness constitutes a reward, especially when social interaction is initiated. Receiving eye-gaze from an attractive face represents a favorable result, leading to enhanced neural responses, whereas failing to make eye-contact with an attractive face is a disappointing outcome, leading to reduced activity in dopaminergic systems. Interestingly, our model suggests that when it comes to mutuality, eye-contact as well as attractiveness play an important role for mate choice. We further argue that perceived attractiveness as well as mutual eye-contact contribute to mate choice individually as they were not related to each other.

Concerning sex differences in gaze behavior, Rogers et al. (2018) report that they found no association between sex and the proportion of gaze toward the eyes or mouth, which we can mostly replicate. When looking at sex differences in gaze behavior within our sample, we found no differences between male and female subjects except the gaze duration toward the eyes and the number of fixations on the head (see Table 3). Other studies applying dual eye-tracking in natural interactions (e.g., Broz et al., 2012; MacDonald & Tatler, 2018; Mayrand et al., 2023) do not report any results on sex differences in gaze behavior.

### Limitations and Future Directions

Although a strength of our research is its ecological validity, a limitation might be its generalizability. Our sample for this study was relatively small, although we analyzed 240 dates; due to the smaller sample size, we could not include a random intercept for the dating partner in our statistical models, which might have yielded additional insights. Moreover, eye-directed gaze produces different effects in an internally-oriented as compared to an externally-oriented culture (e.g., Akechi et al., 2013); thus, the generalizability of our results is limited and should be replicated in different cultures. Second, for several reasons we worked with pre-assigned dating partners. With a round-robin-design it would be possible to investigate the choice behavior of both dating partners, as they would have the same number of speed dates. Third, individuals are further prone to alter their natural gaze behavior while wearing eye-tracking glasses, as knowing that their gaze is being monitored makes them feel more self-conscious about where they are looking at (Foulsham et al., 2011). The effect of being tracked is thus likely to have an impact on our subjects’ gaze behavior (Risko & Kingstone, 2011), ultimately leading to more or less eye-contact than they would naturally exhibit. Fourth, the face and eyes areas within the visual field are rather small and close to each other. Therefore, both the eye-tracking and coding may not be perfectly accurate. Last, results must be interpreted carefully, as gaze behavior might be directly influenced by the first impression of a dating partner (e.g., Willis & Todorov, 2006) and thus adapted appropriately in order to achieve the desired effect e.g., that the other person chooses them.

### Conclusion

By applying dual mobile eye-tracking during romantic interactions, we show that mutual eye-contact predicts mate choice after a 5-min speed-date beyond attractiveness perception. Our results indicate that sharing and receiving eye-contact during romantic interaction has a positive influence on individual mate choice. Our study adds to the sparse body of existing research investigating behavioral indicators of human mate selection.

## Data Availability

The data as well as the statistical analysis code of this study are publicly accessible [https://osf.io/vzx6q/]. Due to privacy protection of our subjects and their dating partners, we do not provide raw eye-tracking material i.e., the videos recorded from the speed dates.

## References

[CR1] Akechi H, Senju A, Uibo H, Kikuchi Y, Hasegawa T, Hietanen JK (2013). Attention to eye contact in the West and East: Autonomic responses and evaluative ratings. PLoS ONE.

[CR2] Apostolou M, Christoforou C (2020). The art of flirting: What are the traits that make it effective?. Personality and Individual Differences.

[CR3] Asendorpf JB, Penke L, Back MD (2011). From dating to mating and relating: Predictors of initial and long-term outcomes of speed-dating in a community sample. European Journal of Personality.

[CR4] Back MD, Penke L, Schmukle SC, Sachse K, Borkenau P, Asendorpf JB (2011). Why mate choices are not as reciprocal as we assume: The role of personality, flirting and physical attractiveness. European Journal of Personality.

[CR5] Baron-Cohen S (1997). Mindblindness: An essay on autism and theory of mind.

[CR6] Broz, F., Lehmann, H., Nehaniv, C. L., & Dautenhahn, K. (2012). Mutual gaze, personality, and familiarity: Dual eye-tracking during conversation. In *2012 IEEE RO-MAN: The 21st IEEE International Symposium on Robot and Human Interactive Communication* (pp. 858–864). IEEE. doi:10.1109/ROMAN.2012.6343859

[CR7] Catron, M. L. (2015, January 9th). To fall in love with anyone, do this. *New York Times*. Retrieved from: https://www.nytimes.com/2015/01/11/style/modern-love-to-fall-in-love-with-anyone-do-this.html

[CR8] Chen T, Helminen TM, Hietanen JK (2017). Affect in the eyes: Explicit and implicit evaluations. Cognition and Emotion.

[CR9] Conty L, Russo M, Loehr V, Hugueville L, Barbu S, Huguet P, Tijus C, George N (2010). The mere perception of eye-contact increases arousal during a word-spelling task. Social Neuroscience.

[CR10] Croes EA, Antheunis ML, Schouten AP, Krahmer EJ (2020). The role of eye-contact in the development of romantic attraction: Studying interactive uncertainty reduction strategies during speed-dating. Computers in Human Behavior.

[CR11] Emery NJ (2000). The eyes have it: The neuroethology, function and evolution of social gaze. Neuroscience and Biobehavioral Reviews.

[CR12] Ewing L, Rhodes G, Pellicano E (2010). Have you got the look? Gaze direction affects judgements of facial attractiveness. Visual Cognition.

[CR13] Fink B, Neave N, Manning JT, Grammer K (2006). Facial symmetry and judgements of attractiveness, health and personality. Personality and Individual Differences.

[CR14] Foulsham T, Walker E, Kingstone A (2011). The where, what and when of gaze allocation in the lab and the natural environment. Vision Research.

[CR15] Fullwood C (2007). The effect of mediation on impression formation: A comparison of face-to-face and video-mediated conditions. Applied Ergonomics.

[CR16] Gobel MS, Kim HS, Richardson DC (2015). The dual function of social gaze. Cognition.

[CR17] Grossmann T (2017). The eyes as windows into other minds: An integrative perspective. Perspectives on Psychological Science.

[CR18] Guy N, Pertzov Y (2023). The robustness of individual differences in gaze preferences toward faces and eyes across face-to-face experimental designs and its relation to social anxiety. Journal of Vision.

[CR19] Helminen TM, Kaasinen SM, Hietanen JK (2011). Eye contact and arousal: The effects of stimulus duration. Biological Psychology.

[CR20] Hessels RS, Cornelissen THW, Hooge ITC, Kemner C (2017). Gaze behavior to faces during dyadic interaction. Canadian Journal of Experimental Psychology.

[CR21] Hietanen JK (2018). Affective eye-contact: An integrative review. Frontiers in Psychology.

[CR22] Ho S, Foulsham T, Kingstone A (2015). Speaking and listening with the eyes: Gaze signaling during dyadic interactions. PLoS ONE.

[CR23] Jarick M, Bencic R (2019). Eye contact is a two-way street: Arousal is elicited by the sending and receiving of eye gaze information. Frontiers in Psychology.

[CR24] Jauk E, Neubauer AC, Mairunteregger T, Pemp S, Sieber KP, Rauthmann JF (2016). How alluring are dark personalities? The dark triad and attractiveness in speed dating. European Journal of Personality.

[CR25] Kampe KK, Frith CD, Dolan RJ, Frith U (2001). Reward value of attractiveness and gaze. Nature.

[CR26] Kampe KK, Frith CD, Frith U (2003). “Hey John”: Signals conveying communicative intention toward the self activate brain regions associated with “mentalizing”, regardless of modality. Journal of Neuroscience.

[CR27] Kellermann J, Lewis J, Laird JD (1989). Looking and loving: The effects of mutual gaze on feelings of romantic love. Journal of Research in Personality.

[CR28] Kleinke CL (1986). Gaze and eye-contact: A research review. Psychological Bulletin.

[CR29] Kobayashi H, Hashiya K (2011). The gaze that grooms: Contribution of social factors to the evolution of primate eye morphology. Evolution and Human Behavior.

[CR30] Kobayashi H, Kohshima S (1997). Unique morphology of the human eye. Nature.

[CR31] Kuzmanovic B, Georgescu AL, Eickhoff SB, Shah NJ, Bente G, Fink GR, Vogeley K (2009). Duration matters: Dissociating neural correlates of detection and evaluation of social gaze. NeuroImage.

[CR32] Lange BP, Zaretsky E, Schwarz S, Euler HA (2014). Words won’t fail: Experimental evidence on the role of verbal proficiency in mate choice. Journal of Language and Social Psychology.

[CR33] Leder H, Mitrovic A, Goller J (2016). How beauty determines gaze! Facial attractiveness and gaze duration in images of real world sciences. i-Perception.

[CR34] Little AC, Jones BC, Penton-Voak IS, Burt DM, Perrett DI (2002). Partnership status and the temporal context of relationships influence human female preferences for sexual dimorphism in male face shape. Proceedings of the Royal Society of London. Series B: Biological Sciences.

[CR35] Luo S, Zhang G (2009). What leads to romantic attraction: Similarity, reciprocity, security, or beauty? Evidence from a speed-dating study. Journal of Personality.

[CR36] MacDonald RG, Tatler BW (2018). Gaze in a real-world social interaction: A dual eye-tracking study. Quarterly Journal of Experimental Psychology.

[CR37] Maran T, Furtner M, Liegl S, Kraus S, Sachse P (2019). In the eye of a leader: Eye-directed gazing shapes perceptions of leaders’ charisma. The Leadership Quarterly.

[CR38] Maran T, Furtner M, Liegl S, Ravet-Brown T, Haraped L, Sachse P (2021). Visual attention in real-world conversation: Gaze patterns are modulated by communication and group size. Applied Psychology.

[CR39] Maran T, Hoffmann A, Sachse P (2022). Early lifetime experience of urban living predicts social attention in real world crowds. Cognition.

[CR40] Maran T, Moder S, Furtner M, Ravet-Brown T, Liegl S (2020). From self-report to behavior: Mapping charisma onto naturalistic gaze patterns. Personality and Individual Differences.

[CR41] Mason MF, Tatkow EP, Macrae CN (2005). The look of love: Gaze shifts and person perception. Psychological Science.

[CR42] Mayrand F, Capozzi F, Ristic J (2023). A dual mobile eye tracking study on natural eye contact during live interactions. Scientific Reports.

[CR43] Miller GF, Todd PM (1998). Mate choice turns cognitive. Trends in Cognitive Sciences.

[CR44] Mitrovic A, Goller J, Tinio PPL, Leder H (2018). How relationship status and sociosexual orientation influence the link between facial attractiveness and visual attention. PLoS ONE.

[CR45] Mogilski JK, Welling LL (2017). The relative importance of sexual dimorphism, fluctuating asymmetry, and color cues to health during evaluation of potential partners’ facial photographs: A conjoint analysis study. Human Nature.

[CR46] Moore MM (1985). Nonverbal courtship patterns in women: Context and consequences. Ethology and Sociobiology.

[CR47] Economic Times Online and Agencies (2023, February 23rd). Dating patterns of 2023: Millennials find ghosting immature, GenZ wants to explore before getting serious, reveals study. *The Economic Times.* Retrieved from: https://economictimes.indiatimes.com//magazines/panache/dating-patterns-of-2023-millennials-find-ghosting-immature-genz-wants-to-explore-before-getting-serious-reveals

[CR48] Perrett DI, Burt DM, Penton-Voak IS, Lee KJ, Rowland DA, Edwards R (1999). Symmetry and human facial attractiveness. Evolution and Human Behavior.

[CR49] Pisanski K, Oleszkiewicz A, Plachetka J, Gmiterek M, Reby D (2018). Voice pitch modulation in human mate choice. Proceedings of the Royal Society B.

[CR50] R Core Team. (2023). *R: A language and environment for statistical computing.* R Foundation for Statistical Computing, Vienna, Austria. https://www.R-project.org/

[CR51] Renninger LA, Wade TJ, Grammer K (2004). Getting that female glance: Patterns and consequences of male nonverbal behavior in courtship contexts. Evolution and Human Behavior.

[CR52] Rhodes G, Simmons LW, Peters M (2005). Attractiveness and sexual behavior: Does attractiveness enhance mating success?. Evolution and Human Behavior.

[CR53] Risko EF, Kingstone A (2011). Eyes wide shut: Implied social presence, eye tracking and attention. Attention, Perception, & Psychophysics.

[CR54] Risko EF, Richardson DC, Kingstone A (2016). Breaking the fourth wall of cognitive science: Real-world social attention and the dual function of gaze. Current Directions in Psychological Science.

[CR55] Rogers SL, Guidetti O, Speelman CP, Longmuir M, Phillips R (2019). Contact is in the eye of the beholder: The eye contact illusion. Perception.

[CR56] Rogers, S. L., Speelman, C. P., Guidetti, O., & Longmuir, M. (2018). Using dual eye-tracking to uncover personal gaze patterns during social interaction. *Scientific Reports,**8*(1). 10.1038/s41598-018-22726-7 10.1038/s41598-018-22726-7 PMC5844880 29523822

[CR57] Rosenthal GG, Ryan MJ (2022). Sexual selection and the ascent of women: Mate choice research since Darwin. Science.

[CR58] Ryan MJ, Keddy-Hector A (1992). Directional patterns of female mate choice and the role of sensory biases. The American Naturalist.

[CR59] Senju A, Johnson MH (2009). The eye contact effect: Mechanisms and development. Trends in Cognitive Sciences.

[CR60] Symons D (1979). The evolution of human sexuality.

[CR61] Thornhill R, Gangestad SW (1999). Facial attractiveness. Trends in Cognitive Sciences.

[CR62] Tidwell ND, Eastwick PW, Finkel EJ (2013). Perceived, not actual, similarity predicts initial attraction in a live romantic context: Evidence from the speed-dating paradigm. Personal Relationships.

[CR63] Tobii, A. B. (2017). *Eye tracker data quality report: Accuracy, precision and detected gaze under optimal conditions—controlled environment*. Tobii Technology.

[CR64] Todd PM, Penke L, Fasolo B, Lenton AP (2007). Different cognitive processes underlie human mate choices and mate preferences. Proceedings of the National Academy of Sciences.

[CR65] Uono, S., & Hietanen, J. K. (2015). Eye-contact perception in the West and East: A cross-cultural study. *PLoS ONE*, 10. 10.1371/journal.pone.0118094 10.1371/journal.pone.0118094 PMC4340785 25714900

[CR66] Valuch C, Pflüger LS, Wallner B, Laeng B, Ansorge U (2015). Using eye-tracking to test for individual differences in attention to attractive faces. Frontiers in Psychology.

[CR67] Van Straaten I, Holland RW, Finkenauer C, Hollenstein T, Engels RCME (2010). Gazing behavior during mixed-sex interactions: Sex and attractiveness effects. Archives of Sexual Behavior.

[CR68] Varina, R. (2022, July 29th). Let’s get to the bottom of that psychology love eye trick from TikTok. *Cosmopolitan*. Retrieved from: https://www.cosmopolitan.com/sex-love/a40730007/psychology-love-eye-trick-tik-tok/

[CR69] Walsh DG, Hewitt J (1985). Giving men the come-on: Effect of eye-contact and smiling in a bar environment. Perceptual and Motor Skills.

[CR70] Weeden J, Sabini J (2005). Physical attractiveness and health in Western societies: A review. Psychological Bulletin.

[CR71] Williams GP, Kleinke CL (1993). Effects of mutual gaze and touch on attraction, mood and cardiovascular reactivity. Journal of Research in Personality.

[CR72] Willis J, Todorov A (2006). First impressions: Making up your mind after a 100-ms exposure to a face. Psychological Science.

